# Online Measurements during Simulated Atmospheric Aging Track the Strongly Increasing Oxidative Potential of Complex Combustion Aerosols Relative to Their Primary Emissions

**DOI:** 10.1021/acs.estlett.4c00956

**Published:** 2024-12-10

**Authors:** Rico K. Y. Cheung, Jun Zhang, Tiantian Wang, Lisa Kattner, Sophie Bogler, Joseph V. Puthussery, Ru-Jin Huang, Martin Gysel-Beer, Jay G. Slowik, Vishal Verma, André S. H. Prevot, Imad El Haddad, David M. Bell, Robin L. Modini

**Affiliations:** † PSI Center for Energy and Environmental Sciences, Paul Scherrer Institute, 5232 Villigen, Switzerland; ‡ Department of Civil & Environmental Engineering, University of Illinois at Urbana−Champaign, Urbana, Illinois 61801, United States; § Department of Energy, Environmental & Chemical Engineering, Washington University in St. Louis, St. Louis, Missouri 63130, United States; ∥ Institute of Earth and Environment, Chinese Academy of Sciences, Xi’an 710061, China

**Keywords:** oxidative potential, wood burning, coal combustion, photooxidation, ozonolysis

## Abstract

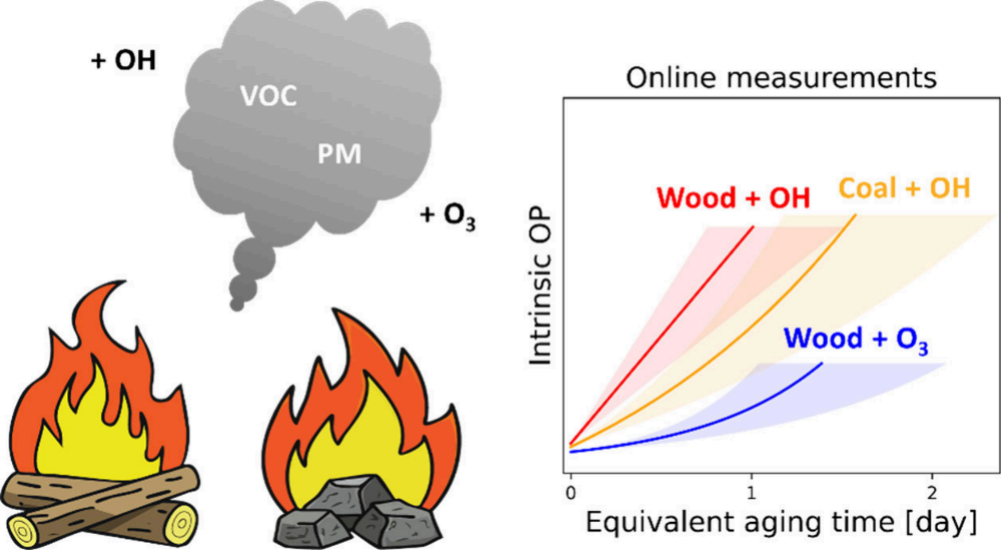

Oxidative potential (OP) is increasingly recognized as a more health-relevant metric than particulate matter (PM) mass concentration because of its response to varying chemical compositions. Given the limited research on the OP of complex combustion aerosols, the effects of aging processes on their OP remain underexplored. We used online instruments to track the evolution of OP [via dithiothreitol (DTT) assays] during the aging of wood burning and coal combustion emissions by hydroxyl-radical-driven photooxidation and dark ozonolysis. We observed very substantial increases in the intrinsic OP (OP_m_^DTT^) of complex combustion aerosols (e.g., OP_m_^DTT^ up to 100 pmol min^–1^ μg^–1^ for OH-aged wood burning emissions) within 1 day of equivalent aging. Further analysis in relation to the degree of oxidation revealed a potential for generalizing the OP of carbonaceous aerosols with average carbon oxidation state  values ranging from −1.5 to −0.5 by assuming they have a constant OP_m_^DTT^ value of ∼10 ± 6 pmol min^–1^ μg^–1^. Additionally, we uncovered a strong dependency of OP_m_^DTT^ on both the source/precursor and aging pathway with  above ∼−0.5. OH photooxidation was identified as an exceptionally efficient pathway for generating highly oxidized, multifunctionalized, and DTT-active products, particularly from wood burning emissions.

## Introduction

Exposure to particulate matter (PM) poses a significant global health risk by causing millions of premature deaths annually.^1−4^ Epidemiological studies have revealed the associations between long-term PM_2.5_ exposure and increased mortalities, as well as hospital admissions, due to cardiovascular and respiratory diseases.^5−7^ Laboratory studies have demonstrated that inhaled PM can generate reactive oxygen species (ROS),^8−10^ and the overproduction of ROS in vivo could eventually lead to oxidative stress, one of the possible mechanisms driving the aerosol toxicity.^11,12^ The ability of PM to generate ROS is commonly referred to as oxidative potential (OP), which was found to have associations with various cardiopulmonary health end points in recent studies.^13−16^

Carbonaceous aerosols, which account for a major fraction of ambient PM,^17,18^ have received specific attention because of their chemical complexity and related health implications. These aerosols are directly emitted into the atmosphere from various sources (e.g., combustion processes) or formed as secondary organic aerosols (SOA) through the atmospheric oxidation of bio- or anthropogenic precursors. Atmospheric aging processes alter their chemical and physical properties, as well as OP. Understanding how aging processes affect the OP of different types of carbonaceous aerosols is therefore crucial for predicting their potential health effects across different regions and under varying atmospheric conditions.

Despite numerous laboratory studies investigating the OP of carbonaceous aerosols, most have focused on the SOA generated from single-precursor systems,^19−29^ while fewer have explored complex combustion aerosols.^30−34^ Moreover, significant uncertainty remains because of varied and unquantified exposures to oxidants and the lack of standardized protocols for measuring OP,^35^ which complicates direct comparisons among studies.

In this work, we conducted smog chamber experiments to examine the OP of carbonaceous aerosols as a function of aging [i.e., photooxidation by hydroxyl radicals (OH) and dark ozonolysis]. These experiments focused on (1) SOA derived from α-pinene (a common biogenic precursor also coemitted during wood burning^36,37^) and naphthalene (an anthropogenic precursor often found in various combustion sources^38^) and (2) complex combustion emissions from wood burning and coal combustion. The evolution of OP due to both oxidation of primary PM and the formation of SOA upon aging was captured using an automated system for performing dithiothreitol (DTT) assays,^39^ complemented by other particle- and gas-phase instruments. These provided transient information for more in-depth comparisons of intrinsic DTT activities across different types of carbonaceous aerosols at a given equivalent aging time.

## Methods and Materials

### Experimental Setup

A schematic of the experimental setup is shown in Figure S1. Experiments were conducted in an atmospheric simulation chamber of the Aerosol, Clouds and Trace Gases Research Infrastructure (ACTRIS)^40^ at the Paul Scherrer Institute,^41^ which is an 8 m^3^ (2 × 2 × 2 m, L × W × H) collapsible Teflon bag housed in a temperature-controlled container with UV-A lamps (λ = ∼365 nm) installed at the bottom for the initiation of photochemistry. The chamber is equipped with generators for nitrous acid (HONO as a continuous OH source^41,42^) and ozone (O_3_). Prior to each experiment, the chamber was preconditioned with clean air supplied by a zero air generator (737-250, AADCO Instruments Inc.).

Complex combustion emissions were generated by burning either spruce/pine logs (Würenlingen, Switzerland) in a residential wood stove^43^ or bituminous coals (Gansu, China) in a Chinese coal stove.^44^ The primary emissions were first diluted with an ejector dilutor (180 °C, DI-1000, Dekati Ltd.) and then introduced into the chamber through heated sampling lines (150 °C). For experiments involving single-precursor systems, α-pinene (99%, Sigma-Aldrich) or naphthalene (99%, Sigma-Aldrich) was added into the chamber as SOA precursors using the protocols described in Bell et al.^45^ The actual durations of the experiments varied from 4 to 10 h (Figures S6–S8). Additional information on experimental procedures is provided in Text S1.

### Online OP Measurements of Suspended Particles

A custom-built system was employed to perform DTT assays automatically (Text S2) coupled with a mist chamber (MC) for semicontinuous sample collection and extraction using Milli-Q water.^39^ The sample air was first denuded of trace gases and then mixed with clean makeup air (0–5 L min^–1^) to achieve a total volumetric flow rate of 16.7 L min^–1^ (∼95% collection efficiency in the MC^46^). After every hour of sample collection and extraction, the sample extracts (i.e., containing mostly water-soluble components plus some insoluble components) were subsequently analyzed by the automated system to determine the DTT consumption rates. The hourly measured DTT consumption rates, after blank subtraction, were normalized by the sampled air volume to calculate the extrinsic OP (OP_v_^DTT^; nmol min^–1^ m^–3^) or by the PM mass loading to calculate the intrinsic OP (OP_m_^DTT^; pmol min^–1^ μg^–1^; see examples in Figure S2). Detailed characterization of this system was documented in Puthussery et al.,^39^ and its field deployment has been demonstrated in several previous studies.^47−49^

### Other Particle- and Gas-Phase Measurements

Particle volume size distributions (15–635 nm) were continuously monitored with a home-built scanning mobility particle sizer (SMPS).^50^ Lognormal fitting was applied to the measurements to estimate the overall size distribution (as illustrated in Figure S3). The total PM mass loading used for calculating OP_m_^DTT^ was determined by multiplying the integrated volume concentration of the fitted size distribution with the sampled air volume of the automated OP system, assuming a particle density of 1 g cm^–3^ for all experiments. Samples with PM mass loadings below 10 μg were excluded from subsequent analyses.

Bulk aerosol compositions were measured with a high-resolution time-of-flight aerosol mass spectrometer (HToF-AMS, Aerodyne). The mass spectra were analyzed using the SQUIRREL version 1.63 and PIKA version 1.23 analysis toolkits. Peak fitting was performed for mass-to-charge ratios (*m*/*z*) from 12 to 170. Elemental ratios of bulk organic aerosols (e.g., O/C and H/C) were calculated using the “improved-ambient” method^51^ and utilized to determine the average carbon oxidation state ().^52^

A photoacoustic aerosol absorption spectrometer (PAAS; schnaiTEC)^53^ was used to measure aerosol light absorption. Concentrations of black carbon (BC) in the primary emissions were calculated by dividing the light absorption at 785 nm by a mass absorption cross section of 11 m^2^ g^–1^. These calculations indicated that BC only contributed minor fractions (0.6 to 7%) to the total mass of directly emitted particles (Table S1).

A proton-transfer reaction mass spectrometer (PTR-TOF 8000, Ionicon Analytik) was deployed to monitor volatile organic compounds (VOC) and the decay of *d*_9_-butanol (98%, Cambridge Isotope Laboratories), and an OH tracer was added into the chamber prior to the aging processes. The OH exposure was determined using the method outlined in Text S3 and Barmet et al.^54^ Additionally, gas analyzers (Thermo 49C and APHA-370) were used to monitor O_3_ and total hydrocarbon (THC) concentration throughout the experiments, and O_3_ exposure was calculated as the integral of measured O_3_ concentration over time. The experimental conditions are summarized fully in Table S1. It can be seen in this table that the oxidant concentrations in our experiments were higher by factors of 1 to 9 than assumed atmospheric levels of 1.5 × 10^6^ molecules cm^–3^ for [OH] (24 h global average OH concentration^55^) and 60 ppb^56,57^ for [O_3_].

## Results and Discussion

### α-Pinene- and Naphthalene-Derived SOA

**Figure 1:**
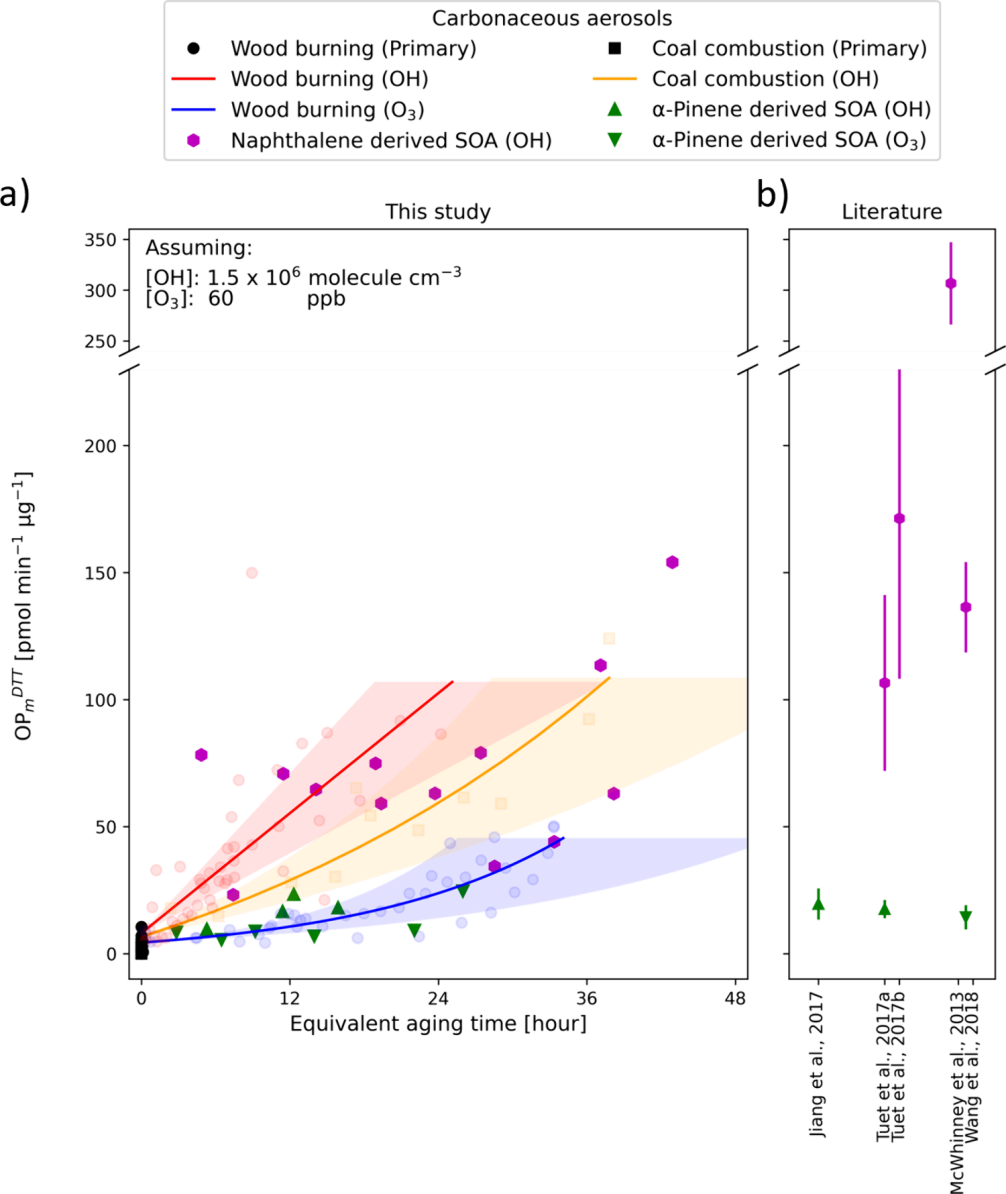
(a) Intrinsic oxidative potential (OP_m_^DTT^) of different carbonaceous aerosols as a function of equivalent aging time, assuming [OH] of 1.5 × 10^6^ molecules cm^–3^ (24 h global average OH concentration^55^) and [O_3_] of 60 ppb,^56,57^ respectively. Solid lines denote the exponential curve fittings for data obtained from the complex combustion experiments. Shaded areas represent fitted results calculated under different assumption of ambient oxidant concentrations ([OH] of 1 × 10^6^ to 2 × 10^6^ molecules cm^–3^ or [O_3_] of 40–80 ppb). (b) Literature values (mean ± 1σ) were harmonized based on the reported sensitivity (Figure S4; Text S4) and the assumed particle density. An unharmonized version of this figure is shown in Figure S5.

**Figure 2:**
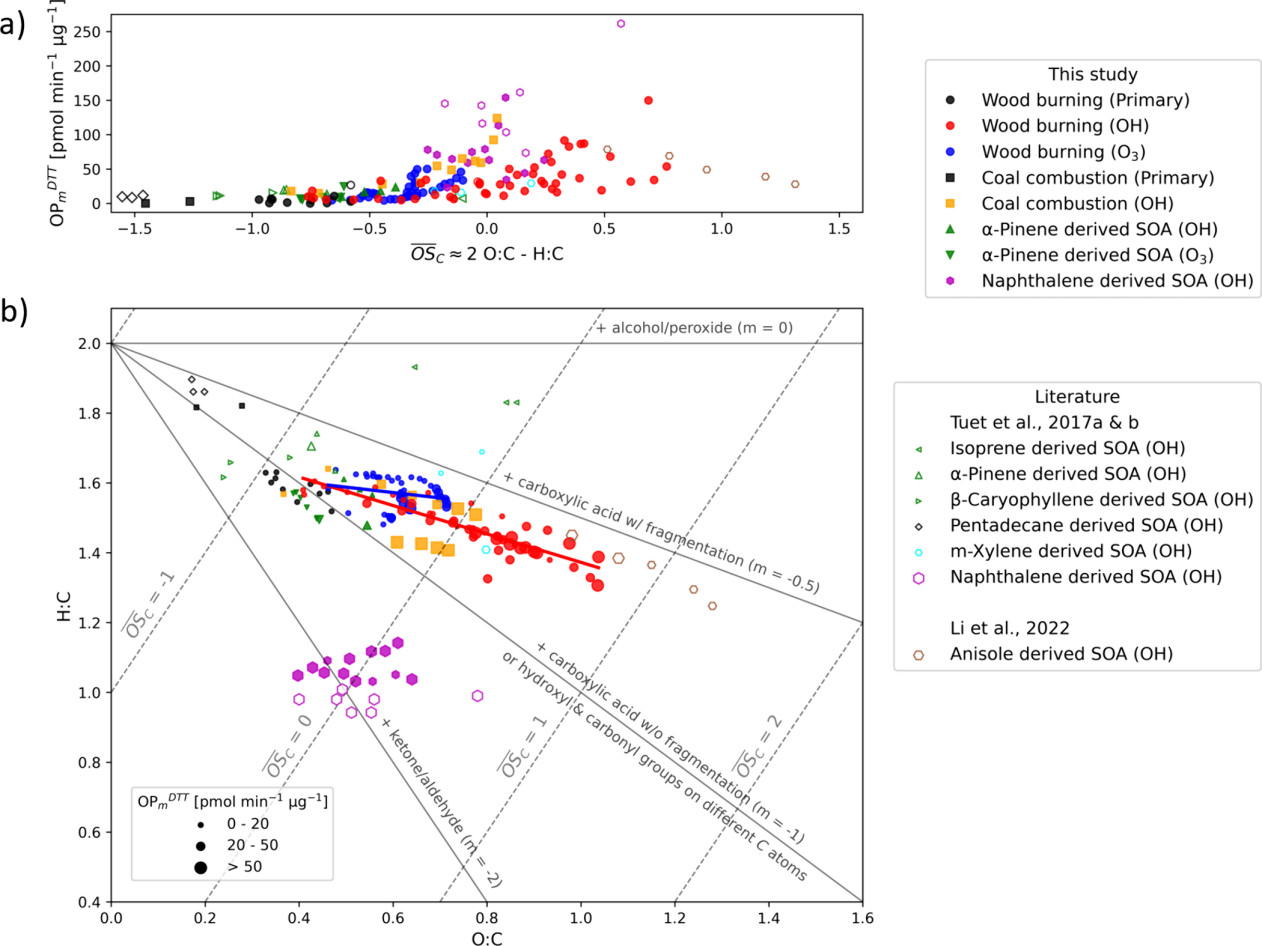
(a) Intrinsic oxidative potential (OP_m_^DTT^) of different carbonaceous aerosols as a function of averaged carbon oxidation state (). (b) Van Krevelen plot for different carbonaceous aerosols. Data points are shaped and colored by the aerosol types. Solid markers denote the data points from this study, while empty markers represent literature values. Data points are sized by intrinsic OP (OP_m_^DTT^). Gray solid lines represent the development of functionalization. A slope of 0 indicates the addition of hydroxyl or hydroperoxy groups. A slope of −0.5 indicates additions of the carboxyl group with fragmentation. A slope of −1 indicates additions of the carboxyl group without fragmentation (or hydroxyl and carbonyl groups on different carbon atoms). A slope of −2 indicates additions of carbonyl groups. Diagonal gray dashed lines denote contours of constant  values.

Given that previous studies have predominantly focused on single-precursor systems, we validated our OP_m_^DTT^ measurements (Figure 1a) against the harmonized literature values (mean ± 1σ; Text S4) for α-pinene- and naphthalene-derived SOA (Figure 2b). Our results show that α-pinene- and naphthalene-derived SOA exhibited distinct redox activity. The OP_m_^DTT^ of α-pinene-derived SOA (either from OH or O_3_) remained low (5–25 pmol min^–1^ μg^–1^) with no obvious trend across the equivalent aging times investigated in this study. In contrast, those of naphthalene-derived SOA were substantially higher (23–150 pmol min^–1^ μg^–1^), despite the large variability that is possibly due to different experimental conditions, as noted in previous studies [e.g., relative humidity,^22^ nitrogen oxides (NOx) levels,^22,26^ and initial precursor concentrations^23^]. Given the limited number of naphthalene experiments conducted, we are unable to draw robust conclusions about potential trends with further aging. Overall, the results from this study are generally consistent with previous studies on single-precursor systems,^19,21−23,25^ including in terms of the magnitude of the measured OP_m_^DTT^ values. The similar magnitude of the values obtained using our online OP instrument with those obtained by offline filter measurements may indicate low contributions of labile species (e.g., organic peroxides) to the DTT activity of these aerosol samples or that the degradation of such labile species does not vary substantially between samples analyzed between 1 h (the present study) and many hours after sample collection (previous offline studies).

### Complex Combustion Aerosols: A Mixture of Primary PM and SOA

Freshly emitted aerosols from wood burning were found to have low OP_m_^DTT^ (4 ± 3 pmol min^–1^ μg^–1^; mean ± 1σ). Figure 1a reveals the OP_m_^DTT^ increased strongly with aging but to varying degrees depending on the aging pathways and the specific types and amounts of SOA formed (Figures S6–S8). Specifically, OH-aged wood burning emissions showed a nearly linear increase in OP_m_^DTT^, reaching about 100 pmol min^–1^ μg^–1^ over 24 h of equivalent aging. In contrast, dark ozonolysis led to a modest increase to approximately 24 pmol min^–1^ μg^–1^ in the same time frame, similar to the levels observed in α-pinene-derived SOA. PTR-TOF data revealed differences between the two aging pathways: a host of aromatic and oxygenated VOCs were consumed in the OH experiments (Figure S9), while monoterpenes and furans were found to be the dominant SOA precursors in O_3_ experiments (Figure S10). OH photooxidation consistently yielded higher OP_m_^DTT^ values than dark ozonolysis at any equivalent aging time probed in this study, likely due to these differences in SOA precursors. Moreover,  increased more rapidly with aging via OH photooxidation than dark ozonolysis (Figure S12), indicating a potential link between OP_m_^DTT^ and the degree of oxidation, which is further explored in the later sections of this Letter.

The increase in OP_m_^DTT^ with aging was also reported in a previous study by Wong et al.^32^ on ambient PM_2.5_ samples affected by wildfires where the majority of the increase occurred within the first 26 h of atmospheric aging. These findings collectively underscore the importance of aging on the OP_m_^DTT^ of wood burning aerosols.

Ambient studies employing source apportionment coupled with multilinear regression models often yielded high model coefficients (interpreted as OP_m_^DTT^) for biomass burning related factors.^58−64^ This contrasts with direct measurements of freshly emitted wood burning aerosols, which exhibit little to no DTT activities, suggesting that the resolved factors in these studies may have undergone some degree of atmospheric aging. However, the extent of aging is often not available in these studies to further explore this hypothesis.

Primary coal combustion aerosols were found to have low OP_m_^DTT^ values (below detection limit −3 pmol min^–1^ μg^–1^), similar to those observed in freshly emitted wood burning aerosols. These measurements are consistent with previous studies showing low OP_m_^DTT^ for primary aerosols from combustion sources.^65,66^ As coal combustion emissions aged under OH photooxidation, a variety of aromatic hydrocarbons reacted to form SOA (Figure S11), and the OP_m_^DTT^ exhibited an upward trend similar to that of the OP_m_^DTT^ for wood burning. In particular, the OP_m_^DTT^ values of aged coal combustions aerosols fell between those for OH photooxidation and dark ozonolysis of wood burning aerosols (Figure 1a).

### Relationships between Intrinsic OP and Carbon Oxidation State

Figure 1 highlights the importance of the source/precursor identity, aging pathway, and exposure time for predicting the OP_m_^DTT^ of carbonaceous aerosols. However, such information is not always available, especially for ambient data. Previous studies have suggested that , a readily measured metric by aerosol mass spectrometer (AMS), could be a proxy to assess the overall aerosol toxicity, as evidenced by the positive correlations between  and various health-relevant metrics.^28,67,68^

In Figure 2a, the OP_m_^DTT^ values from this study, along with the relevant literature values,^22,23,27,33^ are plotted against their corresponding  values. The data reveal an overall increasing trend of OP_m_^DTT^ as a function of . Notably, very minor changes in OP_m_^DTT^ (10 ± 6 pmol min^–1^ μg^–1^) were observed across the  range from approximately −1.5 to −0.5. This suggests that within this  range there is the potential to generalize the OP_m_^DTT^ of carbonaceous aerosols without detailed consideration of specific aerosol types when modeling OP. However, this approach should be taken with caution, and a larger data set is needed to validate these findings with greater certainty. As the oxidation proceeds, OP_m_^DTT^ diverges, thereby necessitating consideration of specific aerosol types for accurately assessing the OP_m_^DTT^ of carbonaceous aerosols with  above ∼−0.5. To further explore the intrinsic OP of carbonaceous aerosols in relation to carbon oxidation state, the same data set is visualized in a van Krevelen diagram^69−71^ (Figure 2b) with data points sized by their corresponding OP_m_^DTT^ values.  in this diagram is represented by the gray, diagonal dashed lines. This allows for better visual separation of the different aerosol types and the tracking of their changes in bulk elemental ratios and the equivalent functionalization.^70,71^

### Source/Precursor-Dependent Relationship

OH photooxidation of biogenic precursors^22^ (i.e., isoprene, α-pinene, and β-caryophyllene) yields SOA that is confined in a narrow space of  from ∼−1 to 0. This SOA shows a minor variability in its OP_m_^DTT^ values. In contrast, SOA derived from anthropogenic precursors (i.e., pentadecane,^22^*m*-xylene,^22^ anisole,^27^ and naphthalene^23^) are sparsely distributed across a broader  space with varying OP_m_^DTT^ values.

OH photooxidation of wood burning and coal combustion emissions appeared to have similar trajectories in the van Krevelen diagram, with their OP_m_^DTT^ values increasing as  increased. However, coal combustion aerosols could reach higher OP_m_^DTT^ values compared to wood burning aerosols at the same , suggesting more DTT-active products being formed from the parent molecules of coal combustion, which are primarily aromatic hydrocarbons (e.g., benzene, toluene, ethylbenzene, xylene, and naphthalene; Figure S11). The oxidized products of aromatic precursors were also suggested as the main contributor to cellular ROS production.^68^ This clearly indicates that the enhancement of OP_m_^DTT^ for complex combustion aerosols upon aging is highly dependent on the fuel type and, ultimately, the SOA precursors.

### Aging-Pathway-Dependent Relationship

To illustrate how OP_m_^DTT^ evolution depends on aging pathways, we consider wood burning aerosols as an example of complex combustion aerosols, which displayed varying degrees and types of oxidation when exposed to different oxidants.

OH photooxidation of wood burning emissions is notably more efficient in producing highly oxidized, multifunctionalized products with over half of the data points centered in the  space from 0 to 1. In contrast, the degree of oxidation for those undergoing dark ozonolysis remained below 0. Additionally, the more oxidized products from OH photooxidation were found to have generally higher OP_m_^DTT^ values than the less oxidized products from dark ozonolysis. Perhaps, the low OP_m_^DTT^ and  for O_3_ aging is not surprising because monoterpenes are a main driver of SOA formation in these experiments, which exhibits low OP_m_^DTT^ and  in single-precursor experiments.

However, data from Figure 2a reveals that at the same level of oxidation (i.e.,  of approximately −0.3), the OP_m_^DTT^ of dark ozonolysis products appeared to be slightly higher than that of OH photooxidation possibly because of the different functionalization pathways as visualized in Figure 2b. OH photooxidation led to net changes in the bulk elemental ratios equivalent to the addition of carboxylic acids with fragmentation (or other equivalent processes), while dark ozonolysis favors the initial addition of alcohols or peroxides of which peroxides have been recognized as a class of DTT-active species.^21,25,72^ This suggests that the type of oxidation could modulate the relationship between OP_m_^DTT^ and .

Overall, these findings highlight that both the degree and type of oxidation of wood burning emissions are subject to the specific aging pathway, which ultimately influences their OP_m_^DTT^. However, it should be noted that these observations may not be generally valid for all aerosol types. For instance, no obvious differences in functionalization and OP_m_^DTT^ were found in α-pinene-derived SOA (either by OH or O_3_) for the  range covered in this study (Figure 2) given the limited number of measurements.

## Implication

In this work, we employed online instruments to track the evolution of intrinsic OP of different carbonaceous aerosols during aging, which revealed significant increases in OP_m_^DTT^ within 1 day of equivalent aging for complex combustion emissions. We explored the relationships between OP_m_^DTT^ and  and uncovered a strong dependency on both the source/precursor and aging pathway for complex combustion emissions with  beyond ∼−0.5. Our results suggest that OH photooxidation is an exceptionally efficient pathway for generating highly oxidized, multifunctionalized, and DTT-active products from complex combustion emissions, particularly wood burning emissions. The observed increases in OP_m_^DTT^ could potentially be explained by multiple chemical processes that occurred simultaneously in our experiments (e.g., gas-phase chemistry, heterogeneous reactions, photoinduced reactions). Additional experimental approaches that selectively examine different fractions of the aged carbonaceous aerosols (both primary and secondary) would be required to better understand the contributions of these processes to the observed changes in OP_m_^DTT^.

Although the trends observed with aging are clear, it is important to note the limitations of our study. We conducted OP measurements on PM suspensions obtained from water mist chamber extraction (i.e., containing mostly water-soluble components plus some insoluble components) using a single acellular assay (DTT). Due to the limited volumes of sample air available in smog chamber experiments, it was not feasible to perform additional measurements with other acellular or cellular assays while achieving the same aging time scales because such measurements typically require high sample flow rates. Even with the instrumentation used here, our aging time scales were limited to up to 2 days of equivalent aging assuming continuous exposure to the oxidants OH and O_3_ (i.e., neglecting diel cycles in the levels of these oxidants). Therefore, our findings do not reflect the full spectrum and dynamics of aerosol OP and toxicity.

Additionally, ambient aerosols are typically more complex than those generated in a controlled laboratory setting and are further influenced by a range of environmental factors that could potentially influence aerosol OP (e.g., temperature, humidity, liquid water content, aerosol acidity, and the presence of mixed particles from diverse sources that may contain high levels of BC and metals). Furthermore, smog chamber artifacts, such as high oxidant levels and differential losses of VOCs and particles to the walls, could also be relevant. All of these variables can potentially impact heterogeneous and particle-phase chemistry and, consequently, overall aerosol OP. Further studies should investigate the influence of variable oxidant levels, diel cycles, and extended aging periods to evaluate the OP of complex combustion aerosols more thoroughly. Finally, we note that future studies are also required to better link the OP of complex combustion aerosols with actual biological impacts, e.g., by incorporating cellular assays that assess other health-relevant end points (e.g., cytotoxicity,^73,75^ intracellular ROS production,^74^ inflammatory biomarkers^67,76,77^) alongside OP measurements.

## Data Availability

The data presented in this study are publicly available on Zenodo (10.5281/zenodo.14204483).

## Supporting Information Available

Additional information on experimental procedures and methods, including the protocol of dithiothreitol (DTT) assay, determination of exposure to oxidant and equivalent aging time, and the harmonization for the literature values (PDF)
