# Supplementary material for: Online Measurements during Simulated Atmospheric Aging Track the Strongly Increasing Oxidative Potential of Complex Combustion Aerosols Relative to Their Primary Emissions
Source: Environ Sci Technol Lett. 2024 Dec 10;12(1):64–72. doi: 10.1021/acs.estlett.4c00956 (PMC11736845; doi:10.1021/acs.estlett.4c00956)
Supplement: Supplementary file 1 — ez4c00956_si_001.pdf [file ez4c00956_si_001.pdf]

**Supporting information**

# Online measurements during simulated atmospheric aging track the strongly increasing oxidative potential of complex combustion aerosols relative to their primary emissions

Rico K.Y. Cheung[1], Jun Zhang[1], Tiantian Wang[1], Lisa Kattner[1], Sophie Bogler[1], Joseph V. Puthussery[3], Ru-Jin Huang[4], Martin Gysel-Beer[1], Jay G. Slowik[1], Vishal Verma[2], André S. H. Prevot[1], Imad El Haddad[1], David M. Bell[1, *], Robin L. Modini[1, *]

[1]PSI Center for Energy and Environmental Sciences, Paul Scherrer Institute, 5232 Villigen PSI, Switzerland
[2]Department of Civil & Environmental Engineering, University of Illinois at Urbana-Champaign, Urbana, Illinois 61801, United States
[3]Department of Energy, Environmental & Chemical Engineering, Washington University in St. Louis, St. Louis, Missouri, 63130, United States
[4]Institute of Earth and Environment, Chinese Academy of Sciences, Xi'an 710061, China
[*] Corresponding authors: david.bell@psi.ch; robin.modini@psi.ch

**Text S1 – 4**

**Table S1 – 2**

**Figure S1 – 12**

**Text S1. Additional information on experimental procedures**

After introducing the complex combustion emissions into the smog chamber, the primary emissions were sampled by the automated OP analyzer for about one hour. During this time, clean humified air was added into chamber as needed. Prior to the start of the aging processes, 1 μL of 9-fold deuterated *n*-butanol ($d_9$-butanol, 98%, Cambridge Isotope Laboratories) was injected into the chamber as a tracer for hydroxyl radicals (OH) in each experiment[1]. Nitrous acid (HONO), serving an OH precursor, was continuously added to the chamber by passing a small flow (1 – 2 L $min^{-1}$) of zero air through the reaction mixture of $H_2SO_4$ (0.1 mM) and $NaNO_2$ (0.2 mM) solutions. Once the concentration of $d_9$-butanol stabilized (5 – 10 mins after injection), the UV lamps were turned on to initiate photochemistry. OP measurements resumed after ~30 minutes of photochemistry. Throughout the experiments, the complex combustion emissions were continuously monitored by other complementary particle- and gas-phase instruments. For the dark ozonolysis experiments, ozone ($O_3$) was introduced into the chamber once the concentration of d9-butanol had stabilized. In the single-precursor system, α-pinene (99%, Sigma Aldrich) or naphthalene (99%, Sigma Aldrich) was added to the chamber as the SOA precursor using the methods outlined in Bell et al.,[2]. Ammonium sulfate ($(NH_4)_2SO_4$) seed particles were added to chamber for some single-precursor experiments (Table S1).

**Text S2. Dithiothreitol (DTT) assay**

After collecting and extracting particles, about 3.5 mL of water extracts from the MC were transferred to a reaction vial (RV) inside a thermomixer (Eppendorf Inc.) using a 10 mL-volume programmable syringe pump (V6, Kloehn lnc). In the RV, 0.5 mL of 1 mM freshly prepared DTT solution (CAS 3483-12-3, >= 99.0% purity, Sigma-Aldrich) and 1 mL of & pH 7.4 potassium phosphate buffer (PBS) solution (CAS 7778-77-0, >=99.0% purity, Sigma-Aldrich; CAS 7758-11-4, ≥98.0% purity, Sigma-Aldrich) were added using another syringe pump with 1-mL internal volume. The mixtures were then incubated at 37 °C for ~1 hour in the thermomixer, which was continuously shaken at 400 rpm to ensure uniformity.

Following the incubation, a 0.1 mL aliquot of the mixtures was transferred to a measurement vial (MV) and mixed with 0.4 mL 0.2mM 5,5'-dithiobis (2-nitrobenzoic acid) (DTNB) solution (CAS 69-78-3, >=98.0% purity, Sigma-Aldrich) and 0.5 mL of Mili-Q water. The remaining DTT then reacted with DTNB to form 2-nitro-5-thiobenzoic acid (TNB) that absorbs light at 412nm. The solution in the MV was diluted 100-fold prior to the absorbance measurements using a UV-Vis detection module, which consisted of a deuterium-tungsten halogen UV-Vis-NIR light source DH-Mini (Ocean Insight Inc.), a liquid waveguide capillary cell LWCC with an optical path length of 100 cm (World Precision Instruments Inc.), and a UV-Vis spectrometer Frame-S (Ocean Insight Inc.). Absorbance was recorded at 412 and 600 nm using a data acquisition software OceanView 2.0 (Ocean Insight Inc.), with the latter used for baseline subtraction. The MV and LWCC were cleaned with Milli-Q water after every measurement. These processes were repeated 5 times within the 1-hour incubation time (t = 10, 20, 30, 40, 50 min). The remaining concentration of DTT left in the RV can be determined using the baseline-subtracted absorbance ($Abs_{412nm}$ – $Abs_{600nm}$) and a calibration curve. The DTT consumption rates (nmol $min^{-1}$) were calculated as the slope of the DTT concentration over time for each sample.

**Text S3. Determination of exposure to oxidant and equivalent aging time**

OH exposure (molecule $cm^{-3}$ s) was estimated based on the decay of $d_9$-butanol and a second volatile organic compound (VOC) measured by a proton-transfer-reaction mass spectrometry (PTR-TOF 8000, Ionicon Analytik) as well as their difference in reactivities with OH radicals[1] (Eq. S1).

$$OH\ exposure(t) = \frac{\ln\frac{[d_9-butanol]_0}{[\mathrm{VOC}\,]_0} - \ln\frac{[d_9-butanol]_t}{[\mathrm{VOC}\,]_t}}{k_{OH,\,\mathrm{d}_9-butanol} - k_{OH,\mathrm{VOC}}} \quad \text{(Equation S1)}$$

where $\frac{[d_9-butanol]_0}{[\mathrm{VOC}\,]_0}$ and $\frac{[d_9-butanol]_t}{[\mathrm{VOC}\,]_t}$ are their concentration ratios at the initial time (defined as the time of perturbation) and at time = $t$ (time after perturbation), respectively; the reaction rate constants with OH radicals for d$_9$-butanol ($k_{OH,\,\mathrm{d}_9-butanol}$) and the second VOC ($k_{OH,\mathrm{VOC}}$). Naphthalene was chosen as the second VOC for most of the experiments, except for α-pinene experiments. Their reactivities with OH radicals at 298 K are provided in Table S2[1,3,4]. If the second VOC is mostly consumed, the OH exposure will be extrapolated under the assumption that the OH concentrations in the chamber remains constant over time. Ozone exposure (ppb h) was calculated as the integral of measured $O_3$ concentration (ppb) over time (Eq. S2).

$$O_3\ exposure(t) = \int_0^t [O_3]\,dt \quad \text{(Equation S2)}$$

Equivalent aging times were calculated as follows:

$$Equivalent\ aging\ time\ (s) = \frac{OH\ exposure(t)}{1.5\times10^6\ molecule\ cm^{-3}} \quad \text{(Equation S3)}$$

or

$$Equivalent\ aging\ time\ (h) = \frac{O_3\ exposure(t)}{60\ ppb} \quad \text{(Equation S4)}$$

**Text S4. Harmonization for the literature values**

Literature values[5–12] (mean ± 1σ) were harmonized based on their reported sensitivity and particle density assumption. Sensitivity was determined by the measured DTT consumption rates as a function of positive control (9,10 phenanthrenequinone or 1,4-naphthoquinone) concentrations reported in different studies relative to those measured in Jiang et al.,[5] with error propagation included (Figure S4). Literature values were then adjusted to align with the relative sensitivity of this study. Additionally, the assumed particle density in these studies was standardized to 1 g cm$^{-3}$ for all types of carbonaceous aerosols. This harmonization process enhances the consistency and comparability of results across different studies. However, it must be stressed that the process is only intended to harmonize sensitivity and density assumption differences between experimental setups used to perform DTT assays. The process is not designed to address differences in the treatment of samples measured by these experimental setups. For example, it does not account for potential differences between online and offline OP measurements, nor does it account for the potential impacts of labile species degradation in offline systems.

Table S1. Experimental conditions

| Experiment | Type | T (°C) | RH (%) | $[PM]_0$[c] (μg $m^{-3}$) | $[BC]_0$[d] (μg $m^{-3}$) | $[THC]_0$[e] (ppm C) | Mean [OH] x$10^6$ (molecule $cm^{-3}$) | OH exposure x$10^{10}$ (molecule $cm^{-3}$ s) | Mean $[O_3]$ (ppb) | $O_3$ exposure (ppb h) |
|---|---|---|---|---|---|---|---|---|---|---|
| 1 | Wood burning + HONO | 20 | < 5 | 455 | 9.6 | 6 | 1.5 | 5.8 | - | - |
| 2 | Wood burning + HONO | 20 | < 5 | 158 | 11 | 3 | 2.0 | 7.3 | - | - |
| 3 | Wood burning + HONO[a] | 20 | < 6 | 64 | 1.4 | 2 | 5.5 | 21 | - | - |
| 4 | Wood burning + HONO[a] | 20 | < 7 | 63 | 0.68 | 1 | 3.4 | 14 | - | - |
| 5 | Wood burning + HONO[a] | 20 | < 8 | 117 | 2.3 | - | 5.6 | 8.0 | - | - |
| 6 | Wood burning + HONO[a] | 20 | < 9 | 344 | 4.6 | - | 1.8 | 6.0 | - | - |
| 7 | Wood burning + $O_3$ | 20 | < 10 | 196 | 7.1 | 6 | - | - | 258 | 2000 |
| 8 | Wood burning + $O_3$ | 20 | < 11 | 158 | 4.4 | 3 | - | - | 180 | 1683 |
| 9 | Wood burning + $O_3$[a] | 20 | < 12 | 67 | 0.45 | 2 | - | - | 97 | 796 |
| 10 | Wood burning + $O_3$[a] | 20 | < 13 | 71 | 0.45 | 4 | - | - | 169 | 1813 |
| 11 | Coal combustion + HONO | 20 | < 14 | 38 | 0.23 | - | 11 | 23 | - | - |
| 12 | Coal combustion + HONO | 20 | < 15 | 76 | 0.59 | - | 11 | 28 | - | - |
| | | | | | | $[VOC]_0$[e] (ppb) | | | | |
| 13 | α-Pinene + HONO[b] | 20 | 45 | - | - | 13 | 11 | 13 | - | - |
| 14 | α-Pinene + HONO | 20 | 70 | - | - | 17 | 7.7 | 13 | - | - |
| 15 | α-Pinene + $O_3$[b] | 20 | < 5 | - | - | 4 | - | - | 230 | 838 |
| 16 | α-Pinene + $O_3$[b] | 20 | 80 | - | - | 3 | - | - | 247 | 1559 |
| 17 | Naphthalene + HONO | 20 | < 5 | - | - | 9 | 14 | 23 | - | - |
| 18 | Naphthalene + HONO | 20 | 70 | - | - | 75 | 8.0 | 23 | - | - |

[a] Only contains emissions during the smouldering phase
[b] Used $(NH_4)_2SO_4$ as seed particles
[c] Subtracted seed particles concentration
[d] Calculated using the aerosol light absorption measured at 785nm and an assumed mass absorption coefficient of 11 $m^2$ $g^{-1}$.
[e] Uncalibrated

Table S2. Reactivities of $d_9$-butanol, Naphthalene and α-Pinene towards OH radicals

| Compound | OH reactivity $k_{OH}$ ($cm^3$ $molecule^{-1}$ $s^{-1}$) | Literature |
|---|---|---|
| $d_9$-butanol | $3.4 \times 10^{-12}$ | Barmet et al. (2012) |
| Naphthalene | $2.3 \times 10^{-11}$ | Calvert et al. (2015) |
| α-Pinene | $5.3 \times 10^{-11}$ | IUPAC 2017 |

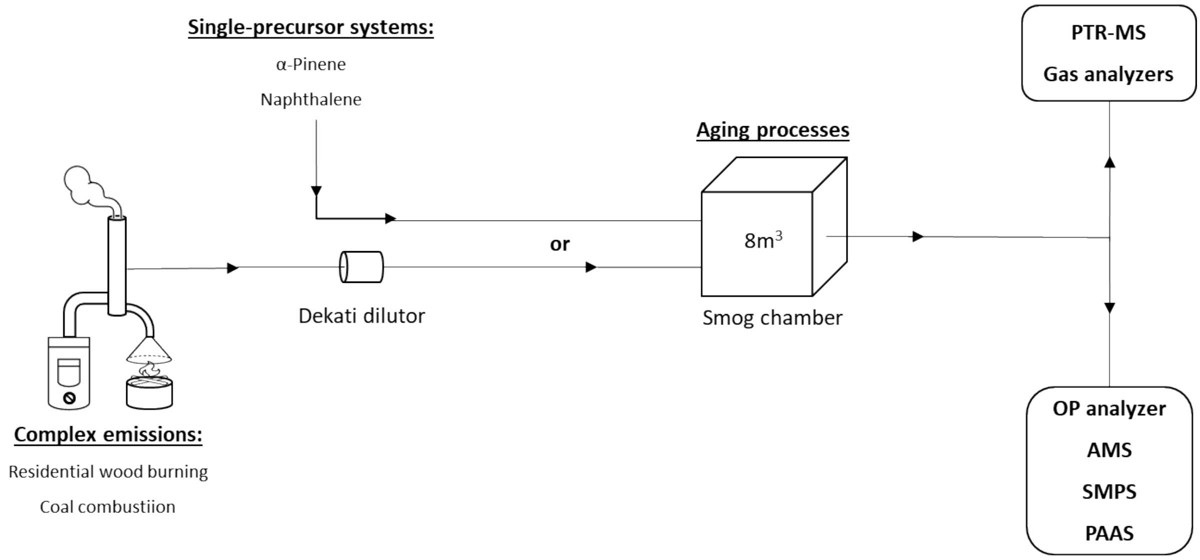


Figure S1. Schematic diagram of experimental setup.

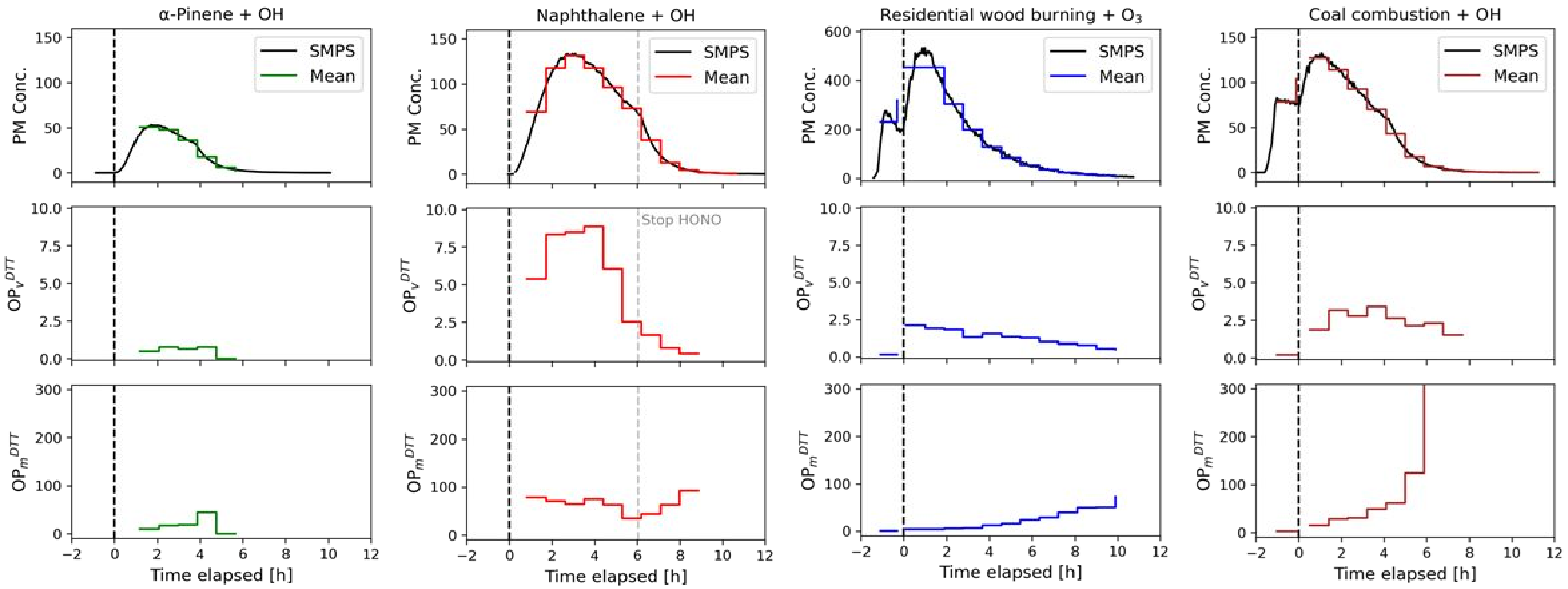


Figure S2. Examples of time series obtained from single-precursor systems and complex combustion experiments showing the evolution of SMPS-measured PM mass concentrations (assuming a particle density of 1 g $cm^{-3}$) in the top panel and the extrinsic OP ($OP_v^{DTT}$) in the middle panel. The intrinsic OP ($OP_m^{DTT}$) shown in the bottom panel were derived from $OP_v^{DTT}$ divided by the mean PM concentrations over the same collection period. The dark dashed lines (defined as t = 0) denote the perturbation time when aging processes were initiated. Samples with PM mass loading less than 10 µg were excluded from the subsequent analyses.

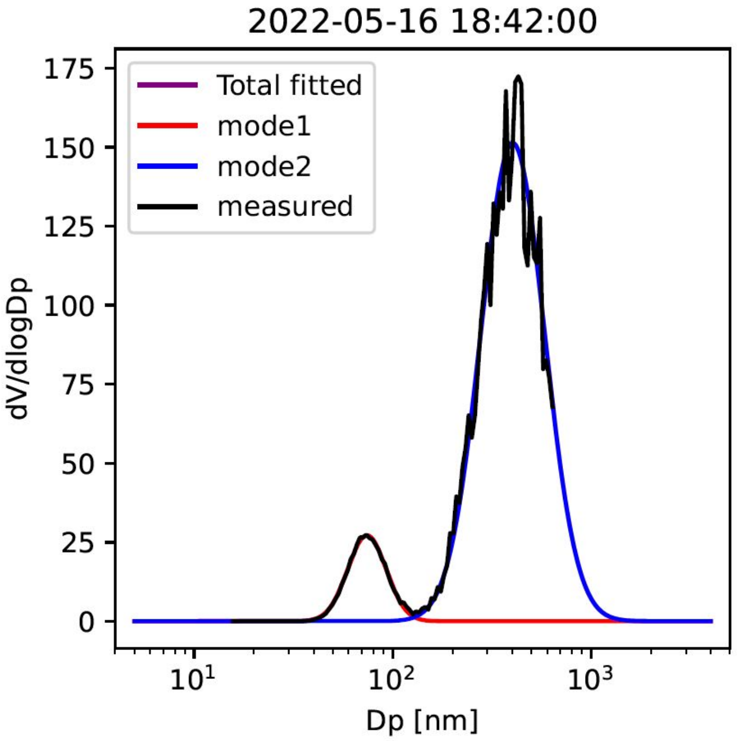


Figure S3. Example of a particle volume size distribution (15 – 635 nm) measured by a SMPS during a wood burning experiment with aging. Lognormal fitting was applied to the data to estimate the overall size distribution, addressing the issue of a considerable fraction of particles exceeding the upper size cut.

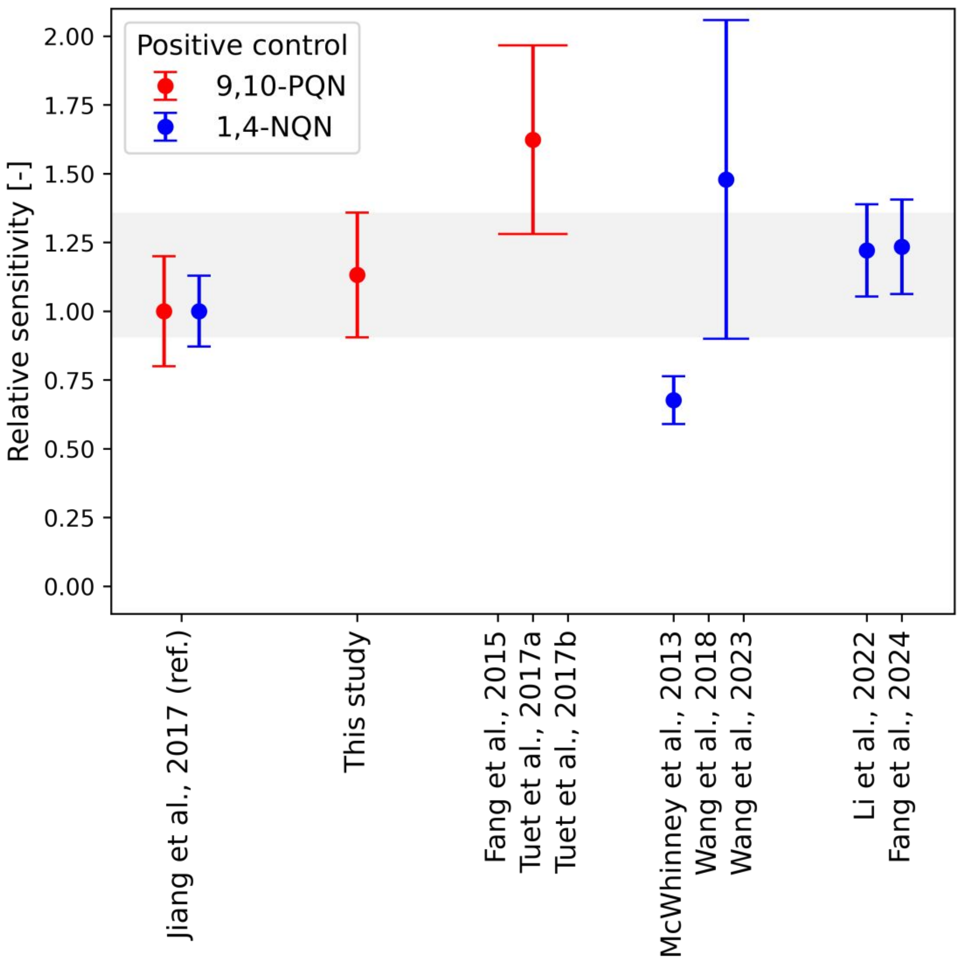


Figure S4. Sensitivity determined by the measured DTT consumption rates as a function of positive control (9,10 phenanthrenequinone or 1,4-naphthoquinone) concentrations reported in different studies relative to those measured in Jiang et al. (2017) with error propagation. Shaded area denotes the range of relative sensitivity of this study. Literature values were then adjusted to align with the relative sensitivity of this study.

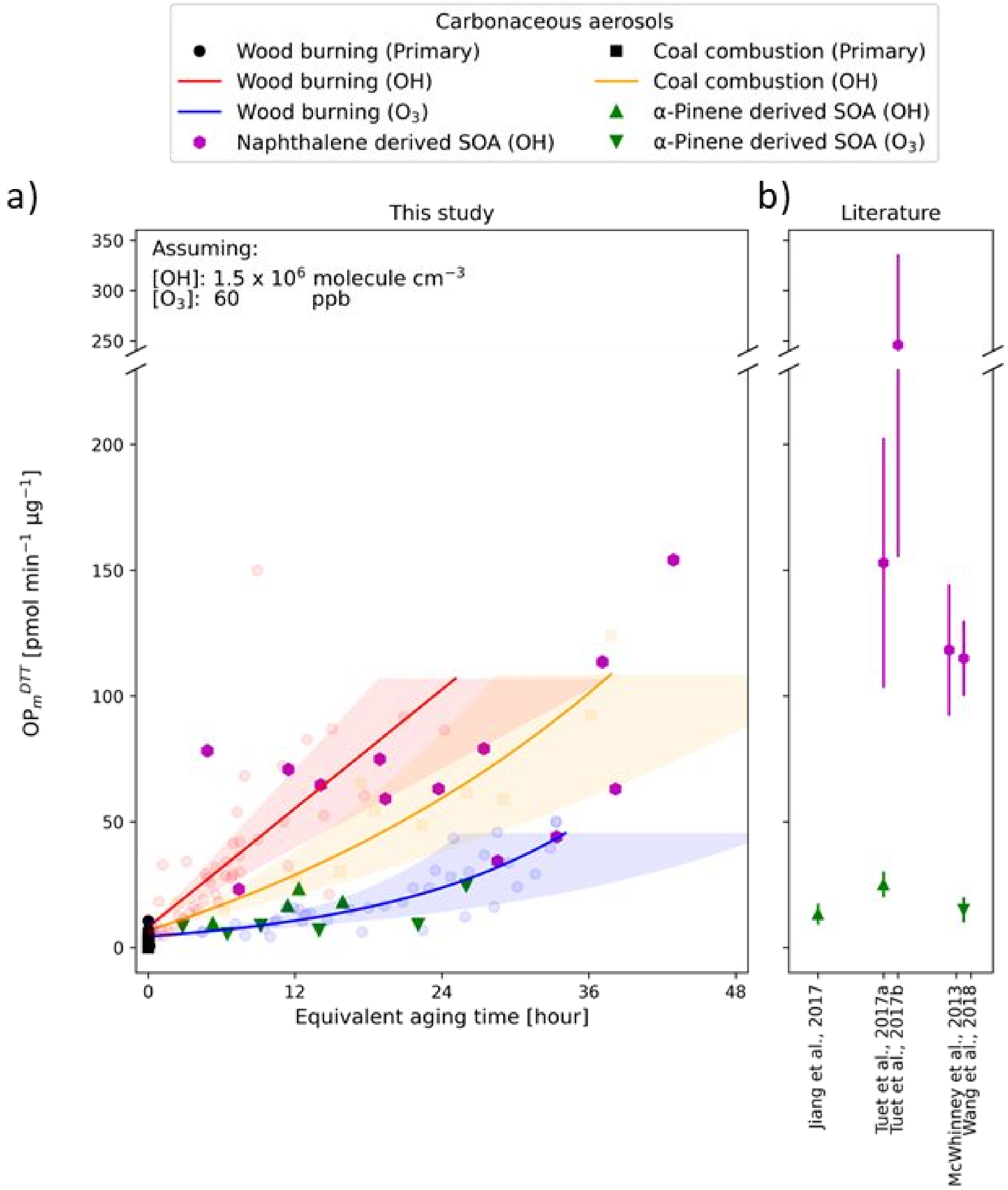


Figure S5. An unharmonized version of fig. 1 (a) Intrinsic oxidative potential ($OP_m^{DTT}$) of different carbonaceous aerosols as a function of equivalent aging time, assuming daily average OH and $O_3$ concentration of 1.5 x $10^6$ molecule $cm^{-3}$ and 60 ppb respectively. Solid lines denote the exponential curve fitting for data obtained from complex combustion experiments while shaded areas represent the fitted results with assuming OH concentrations of 1 x $10^6$ – 2 x $10^6$ molecule $cm^{-3}$ or $O_3$ concentrations of 40 – 80 ppb. (b) Literature values (mean ± 1σ).

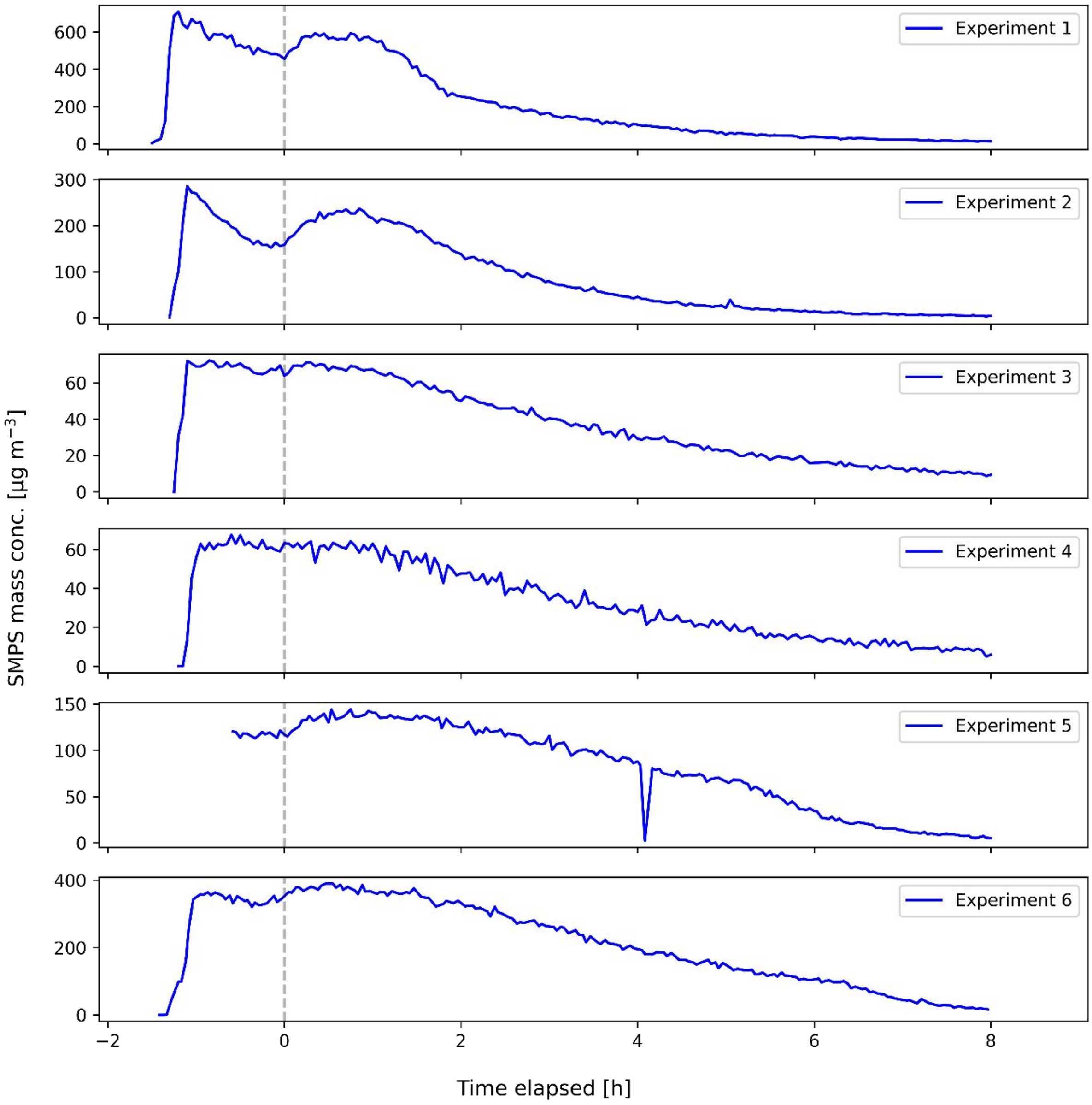


Figure S6. Time series of PM mass concentration for experiment 1 to 6. The dark dashed lines (defined as t = 0) denote the perturbation time when aging processes were initiated.

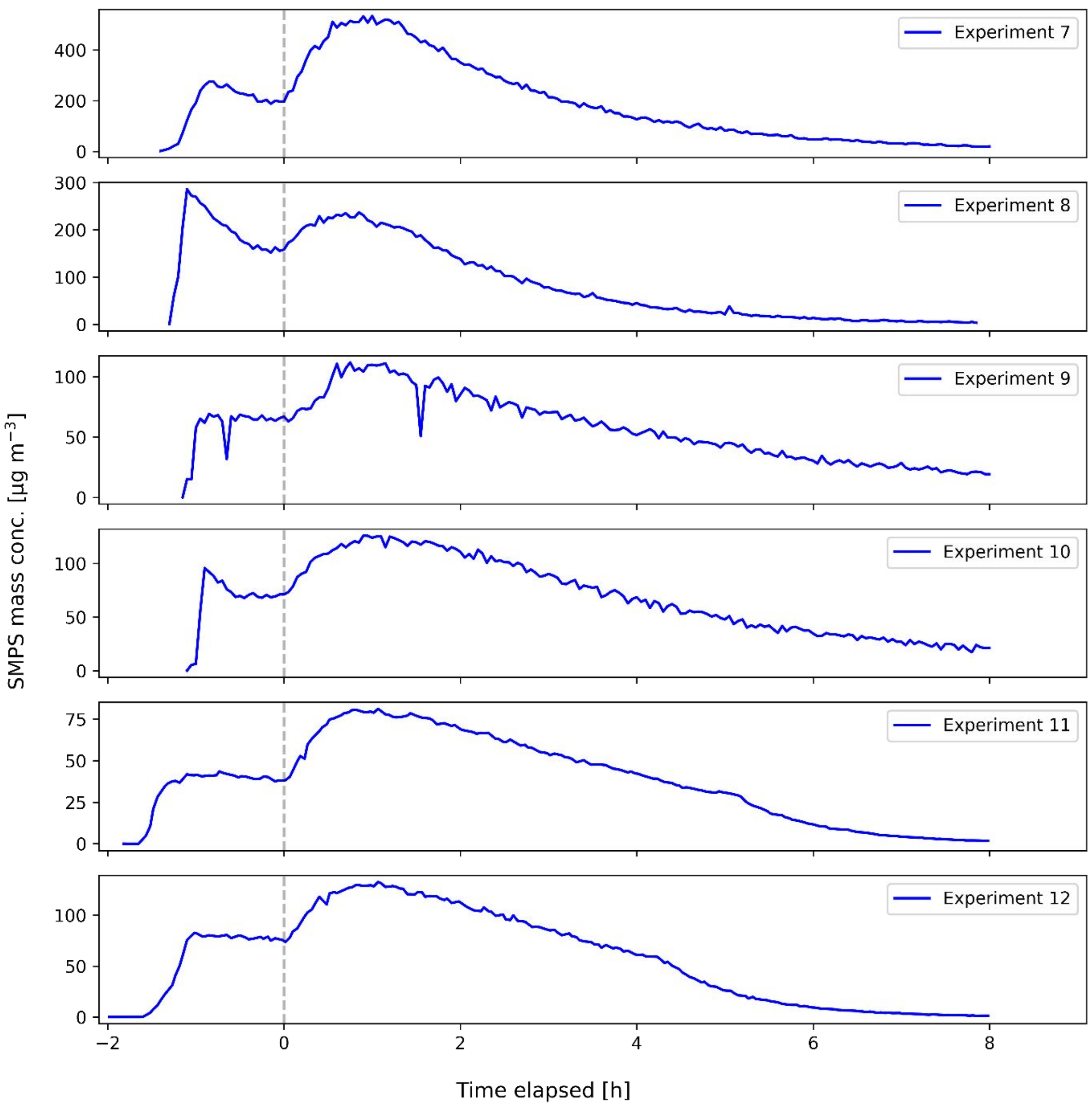


Figure S7. Time series of PM mass concentration for experiment 7 to 12. The dark dashed lines (defined as t = 0) denote the perturbation time when aging processes were initiated.

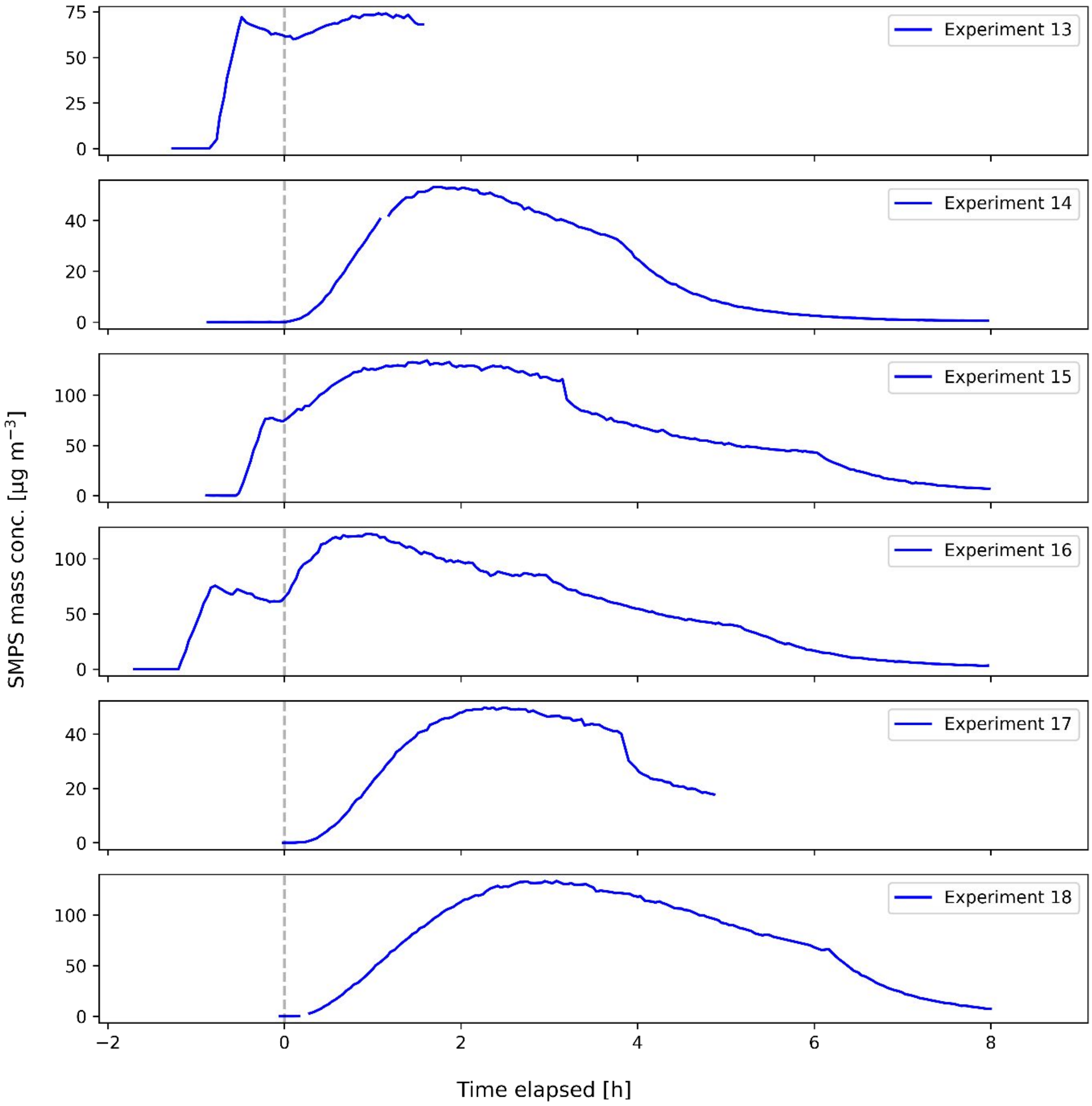


Figure S8. Time series of PM mass concentration without seed particle subtraction for experiment 13 to 18. The dark dashed lines (defined as t = 0) denote the perturbation time when aging processes were initiated.

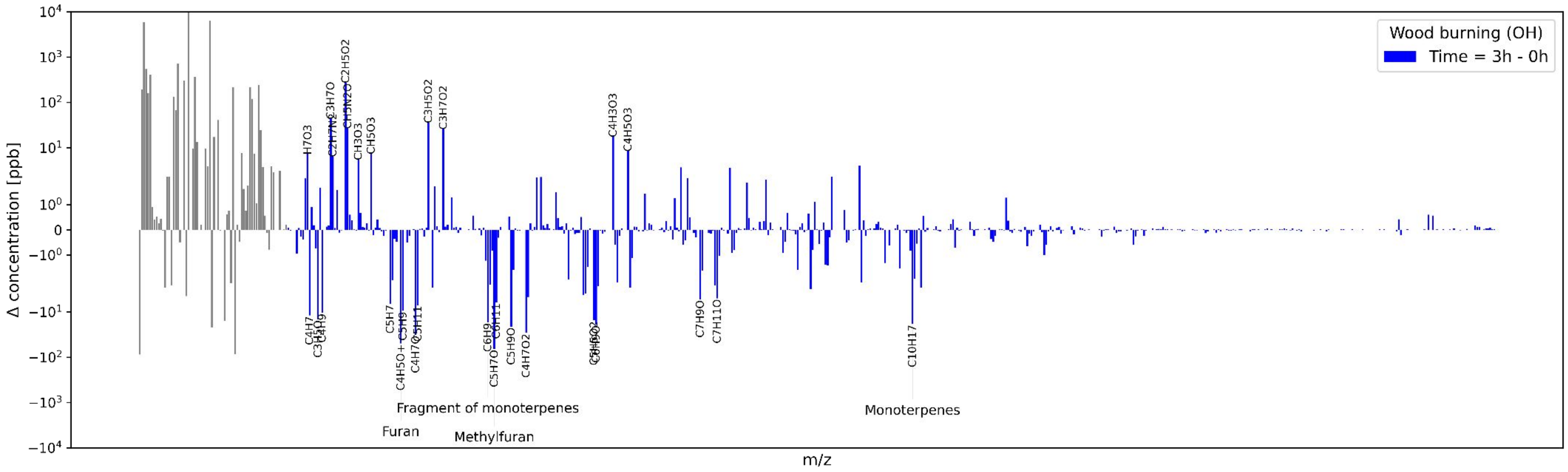


Figure S9. Example of the mass spectral difference between the VOCs measured by PTR-ToF at time = 3h and 0h for OH photooxidation of wood burning emissions. The concentrations have been corrected for the dilution. The m/z from 0 – 50 are shown in grey.

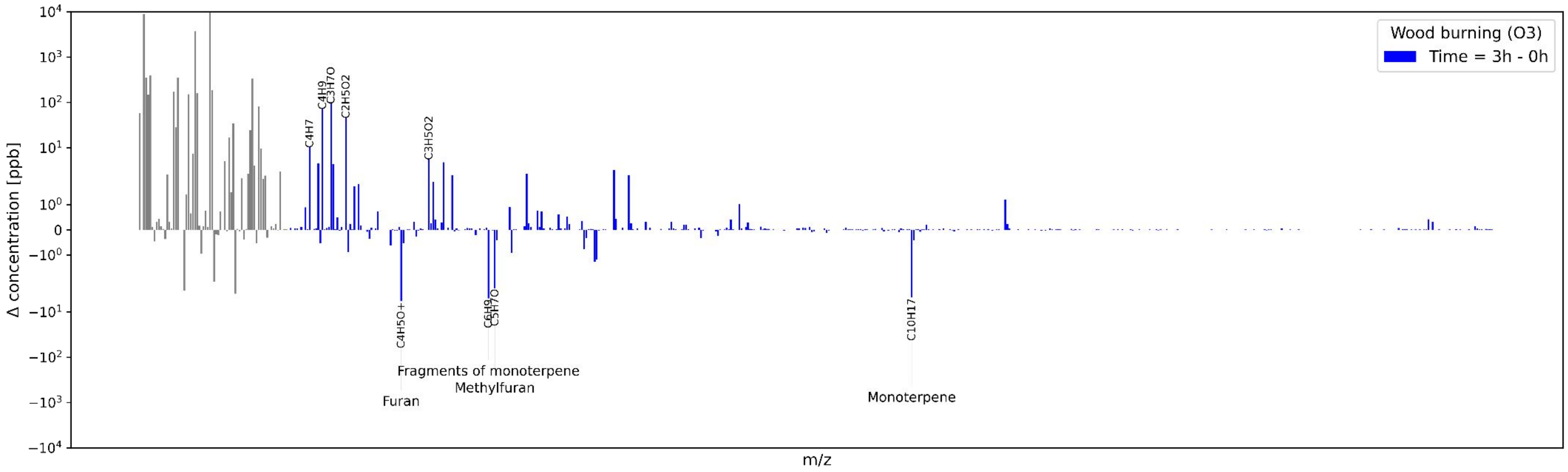


Figure S10. Example of the mass spectral difference between the VOCs measured by PTR-ToF at time = 3h and 0h for dark ozonolysis of wood burning emissions. The concentrations have been corrected for the dilution. The m/z from 0 – 50 are shown in grey.

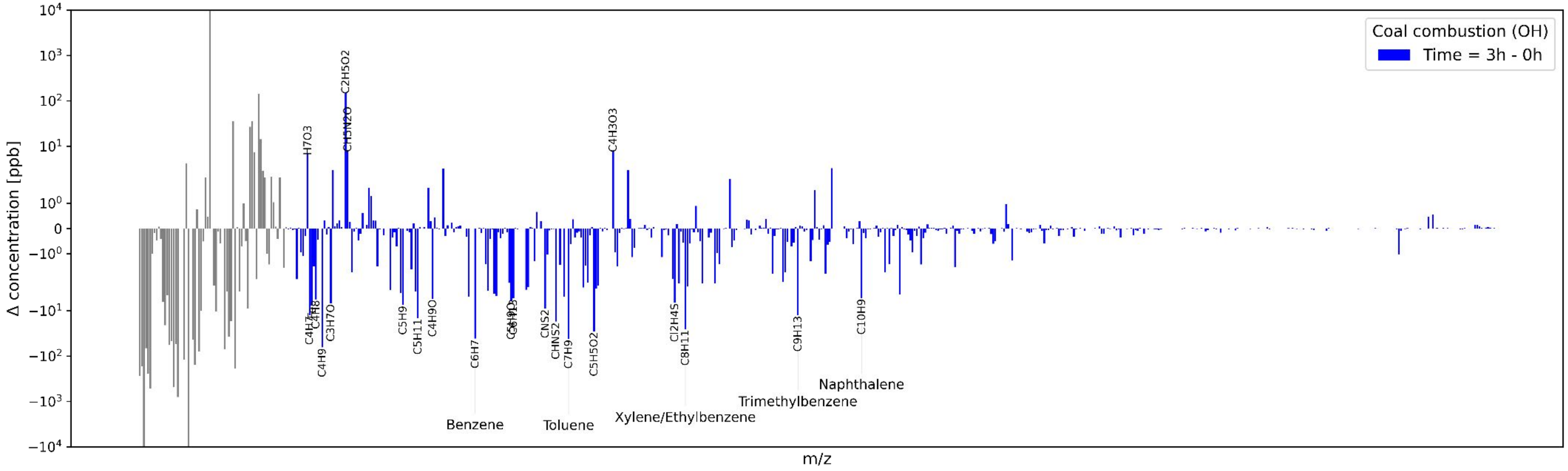


Figure S11. Example of the mass spectral difference between the VOCs measured by PTR-ToF at time = 3h and 0h for OH photooxidation of coal combustion emissions. The concentrations have been corrected for the dilution. The m/z from 0 – 50 are shown in grey.

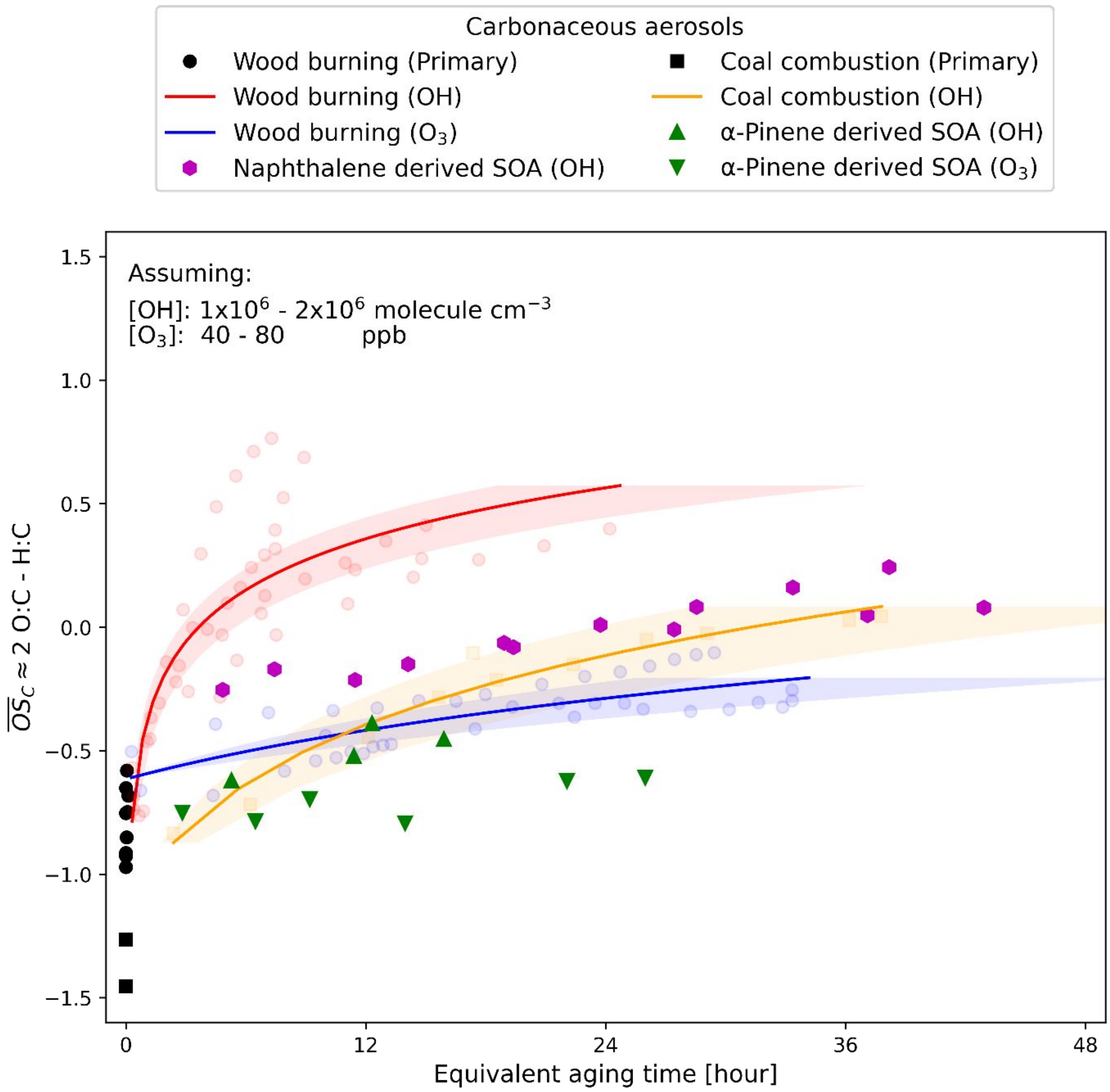


Figure S12. Averaged carbon oxidation state ($\overline{OS_C}$) different carbonaceous aerosols as a function equivalent aging time, assuming daily average OH and $O_3$ concentration of 1.5 x $10^6$ molecule $cm^{-3}$ and 60 ppb respectively. Solid lines denote the exponential curve fitting for data obtained from complex combustion experiments while shaded areas represent the fitted results with assuming OH concentrations of 1 x $10^6$ – 2 x $10^6$ molecule $cm^{-3}$ or $O_3$ concentrations of 40 – 80 ppb.

## References


(1) Barmet, P.; Dommen, J.; DeCarlo, P. F.; Tritscher, T.; Praplan, A. P.; Platt, S. M.; Prévôt, A. S. H.; Donahue, N. M.; Baltensperger, U. OH Clock Determination by Proton Transfer Reaction Mass Spectrometry at an Environmental Chamber. *Atmospheric Measurement Techniques* **2012**, *5* (3), 647–656. https://doi.org/10.5194/amt-5-647-2012.

(2) Bell, D. M.; Cirtog, M.; Doussin, J.-F.; Fuchs, H.; Illmann, J.; Muñoz, A.; Patroescu-Klotz, I.; Picquet-Varrault, B.; Ródenas, M.; Saathoff, H. Preparation of Experiments: Addition and In Situ Production of Trace Gases and Oxidants in the Gas Phase. In *A Practical Guide to Atmospheric Simulation Chambers*; Doussin, J.-F., Fuchs, H., Kiendler-Scharr, A., Seakins, P., Wenger, J., Eds.; Springer International Publishing: Cham, 2023; pp 129–161. https://doi.org/10.1007/978-3-031-22277-1_4.

(3) Calvert, J. G.; Orlando, J. J.; Stockwell, W. R.; Wallington, T. J.; Calvert, J. G.; Orlando, J. J.; Stockwell, W. R.; Wallington, T. J. *The Mechanisms of Reactions Influencing Atmospheric Ozone*; Oxford University Press: Oxford, New York, 2015.

(4) IUPAC. *Task Group on Atmospheric Chemical Kinetic Data Evaluation*. https://iupac.aeris-data.fr/en/home-english/ (accessed 2024-06-26).

(5) Jiang, H.; Jang, M.; Yu, Z. Dithiothreitol Activity by Particulate Oxidizers of SOA Produced from Photooxidation of Hydrocarbons under Varied NO<Sub><I>X</I></Sub> Levels. *Atmos. Chem. Phys.* **2017**, *17* (16), 9965–9977. https://doi.org/10.5194/acp-17-9965-2017.

(6) Tuet, W. Y.; Chen, Y.; Xu, L.; Fok, S.; Gao, D.; Weber, R. J.; Ng, N. L. Chemical Oxidative Potential of Secondary Organic Aerosol (SOA) Generated from the Photooxidation of Biogenic and Anthropogenic Volatile Organic Compounds. *Atmos. Chem. Phys.* **2017**, *17* (2), 839–853. https://doi.org/10.5194/acp-17-839-2017.

(7) Tuet, W. Y.; Chen, Y.; Fok, S.; Gao, D.; Weber, R. J.; Champion, J. A.; Ng, N. L. Chemical and Cellular Oxidant Production Induced by Naphthalene Secondary Organic Aerosol (SOA): Effect of Redox-Active Metals and Photochemical Aging. *Sci Rep* **2017**, *7* (1), 15157. https://doi.org/10.1038/s41598-017-15071-8.

(8) McWhinney, R. D.; Zhou, S.; Abbatt, J. P. D. Naphthalene SOA: Redox Activity and Naphthoquinone Gas–Particle Partitioning. *Atmos. Chem. Phys.* **2013**, *13* (19), 9731–9744. https://doi.org/10.5194/acp-13-9731-2013.

(9) Wang, S.; Ye, J.; Soong, R.; Wu, B.; Yu, L.; Simpson, A. J.; Chan, A. W. H. Relationship between Chemical Composition and Oxidative Potential of Secondary Organic Aerosol from Polycyclic Aromatic Hydrocarbons. *Atmos. Chem. Phys.* **2018**, *18* (6), 3987–4003. https://doi.org/10.5194/acp-18-3987-2018.

(10) Wang, S.; Gallimore, P. J.; Liu-Kang, C.; Yeung, K.; Campbell, S. J.; Utinger, B.; Liu, T.; Peng, H.; Kalberer, M.; Chan, A. W. H.; Abbatt, J. P. D. Dynamic Wood Smoke Aerosol Toxicity during Oxidative Atmospheric Aging. *Environ. Sci. Technol.* **2023**, *57* (3), 1246–1256. https://doi.org/10.1021/acs.est.2c05929.

(11) Li, C.; Misovich, M. V.; Pardo, M.; Fang, Z.; Laskin, A.; Chen, J.; Rudich, Y. Secondary Organic Aerosol Formation from Atmospheric Reactions of Anisole and Associated Health Effects. *Chemosphere* **2022**, *308*, 136421. https://doi.org/10.1016/j.chemosphere.2022.136421.

(12) Fang, Z.; Lai, A.; Dongmei Cai; Chunlin Li; Carmieli, R.; Chen, J.; Wang, X.; Rudich, Y. Secondary Organic Aerosol Generated from Biomass Burning Emitted Phenolic Compounds: Oxidative Potential, Reactive Oxygen Species, and Cytotoxicity. *Environ. Sci. Technol.* **2024**, *58* (19), 8194–8206. https://doi.org/10.1021/acs.est.3c09903.
